# A seven-dimensional quantum dynamics study of the dissociative chemisorption of H_2_O on Cu(111): effects of azimuthal angles and azimuthal angle-averaging†
†Electronic supplementary information (ESI) available: Details of the new full-dimensional potential energy surface and seven-dimensional quantum dynamics calculations. See DOI: 10.1039/c5sc03689e


**DOI:** 10.1039/c5sc03689e

**Published:** 2015-11-25

**Authors:** Tianhui Liu, Zhaojun Zhang, Bina Fu, Xueming Yang, Dong H. Zhang

**Affiliations:** a Department of Chemical Physics , University of Science and Technology of China , Hefei , China 230026; b State Key Laboratory of Molecular Reaction Dynamics and Center for Theoretical and Computational Chemistry , Dalian Institute of Chemical Physics , Chinese Academy of Sciences , Dalian , China 116023 . Email: bina@dicp.ac.cn ; Email: zhangdh@dicp.ac.cn

## Abstract

We report the first seven-dimensional quantum dynamics study for the dissociative chemisorption of H_2_O on Cu(111) using the time-dependent wave-packet approach, based on an accurate nine-dimensional potential energy surface (PES), which is newly developed by neural network fitting to ∼80 000 density functional theory points. This seven-dimensional quantum model allows the examination of the influence of azimuthal angles and also the investigation of the quantitative relationship between the seven-dimensional (7D) dissociation probabilities and those results calculated by the six-dimensional (6D) model with the flat surface approximation. The reactivity strongly depends on the azimuthal rotations due to different barrier heights. Very large differences are seen between the 7D dissociation probabilities and the 6D results with fixed azimuthal angles, at different fixed sites of impact, indicating that the 6D model by neglecting the azimuthal rotation can introduce substantial errors in calculating dissociation probabilities and the 7D quantum dynamics is essential to investigate the dissociation process. A new azimuthal angle-averaging approach is proposed that the 7D dissociation probability can be well reproduced by averaging 6D results over 18 azimuthal angles, in particular in low kinetic energy regions.

## Abstract 2

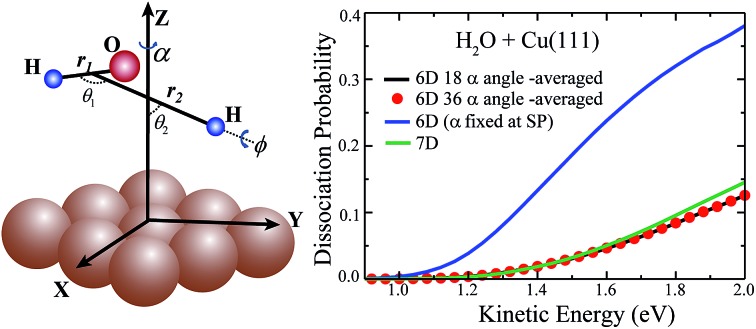
A seven-dimensional quantum dynamics study for the dissociative chemisorption of H_2_O on Cu(111) is reported, using the time-dependent wave-packet approach.

## 

The interaction of molecular species with metal surfaces plays a pivotal role in understanding heterogeneous catalysis. Dissociative chemisorption, which features the surface assisted dissociation of a gas-phase molecule into chemisorbed products, is often the initial and rate-limiting step in many industrial heterogeneous processes such as steam reforming, ammonia synthesis and the water–gas shift reaction.^1^ Due to its significance, tremendous progress has been made for the dissociative chemisorption of molecules in the past two decades.^2^–^16^

A large number of molecular beam experiments have revealed the nonstatistical dynamics including mode specificity and bond selectivity in dissociative chemisorption processes.^3^–^6^,^9^,^12^–^16^ However, theoretically it is still challenging to investigate those processes at a full-dimensional quantum mechanical level, in particular for the polyatomic molecules dissociating on surfaces, mainly owing to the difficulties in constructing reliable high-dimensional potential energy surfaces (PESs) and developing quantum mechanical methodologies.^17^ Such full-dimensional quantum dynamics calculations were limited to diatomic molecules dissociating on metal surfaces,^18^–^32^ such as state-of-the-art dynamics calculations for prototypical surface reactions involving H_2_, although many problems are not yet resolved, *e.g.*, the effect of phonons.^18^,^19^,^22^,^27^,^32^

Recently, much more attention has been focused on polyatomic molecules, such as water and methane. The *ab initio* molecular dynamics approach, which computes the potential energies and forces along the trajectory on the fly, has been employed to study dissociative chemisorption dynamics.^33^–^35^ Nonetheless, such calculations are computationally too expensive, and they also neglect quantum effects. Although quasiclassical trajectory (QCT) calculations based on global PESs are considerably less expensive and shed valuable light on the dynamics,^36^ the accurate full quantum characterization of dissociative chemisorption of polyatomic molecules is highly desirable in view of potentially important quantum effects such as tunneling, zero-point energy and resonances.

The dissociative chemisorption of water on transition-metal surfaces is an essential fundamental step of steam reforming and the water–gas shift reaction.^37^ The dynamics of this gas-surface reaction is quantum mechanical in nature due to large zero-point energies and high barriers, where tunneling is important. Since a total of 9 degrees (9D) of freedom on the rigid surface should be considered, a fully coupled 9D quantum treatment of this gas-surface reaction is still formidable. Tiwari and co-workers employed the pseudo-diatomic molecule model to investigate the dynamics for the H_2_O/Cu(111) system, which was based on a three dimensional LEPS PES.^38^ Guo and co-workers carried out six-dimensional (6D) quantum dynamics calculations to investigate the mode specificity and bond selectivity for the systems of H_2_O/HOD on a rigid flat Cu(111) surface, based on their 6D PESs fitted by permutationally invariant polynomials.^39^–^41^ The lateral coordinates (X and Y) and the azimuthal angle are fixed at the transition state geometry, without considering the effect of impact sites and surface corrugation. The first molecular beam experiment on the dissociative chemisorption of D_2_O on Ni(111) revealed a strong enhancement in reactivity upon excitation of the antisymmetric stretch of D_2_O.^16^ The theoretical results obtained using the six-dimensional quantum approach by Guo and co-workers discussed above semiquantitatively reproduced the observed mode specificity.^16^ Recently, Farjamnia and Jackson employed a fully quantum model to investigate the dissociative chemisorption of water on Ni(111),^42^ based on the reaction path Hamiltonian (RPH).^43^ Although the RPH approach is full-dimensional, it is approximate and only accurate near the minimum energy path. A quantitative description of a gas-surface reaction can only be achieved with a fully coupled quantum mechanical approach based on an accurate global PES.

Very recently, Jiang and Guo performed quasiclassical trajectory and (6 + 1)D quantum dynamics calculations to explore the dynamics of the dissociative chemisorption of D_2_O on rigid Ni(111), using a new nine-dimensional PES developed by a permutation invariant polynomial-neural network (PIP-NN) method.^44^,^45^ In their (6 + 1)D quantum model, only six coordinates were treated explicitly, but the azimuthal angle was fixed at the value of the corresponding saddle point of specific sites, thus the flat surface approximation was invoked. The influence of impact sites and incident angles was examined, indicating that the reactivity depends on the site of impact, which is determined by the topography of the PES, but not the static barrier height alone. This suggests that the simple energy shifting site-averaging model employed in many previous studies,^35^,^46^ based on the assumption that dissociation probabilities at different impact sites have the same energy dependence, but varying with the barrier height, should not be quite reliable. A more rigorous site-averaging treatment is to average reduced-dimensional fixed-site dissociation probabilities over multiple symmetric sites, as was reported recently by our group for HCl/DCl dissociating on Au(111)^28^,^29^ and H_2_ dissociating on Cu(111).^27^ The validity of this site-averaging approximation should generally hold in many molecule–surface interactions such as the dissociative chemisorption of water on metal surfaces. Therefore, one can eventually obtain the fully coupled quantum dynamical probabilities by averaging the seven-dimensional (7D) fixed-site results, instead of directly doing 9D calculations, which is currently computationally more formidable.

In this article, we report the first 7D quantum dynamics calculations for the dissociative chemisorption of H_2_O on a corrugated, rigid Cu(111) surface, which are represented in molecular coordinates (shown in Fig. 1) by a 7D time-dependent wave packet method. The translational coordinates of the H_2_O moiety in the *XY* plane are fixed at a specific site of impact. The first globally accurate PES in full 9 dimensions for water dissociative chemisorption on Cu(111) is developed by neural network (NN) fitting to roughly 80 000 DFT energy points, employing the *C*_3v_ symmetry (the *P*3*m*1 plane group symmetry^47^) of the Cu(111) surface (shown in Fig. S1†). This fit results in an overall very small root mean square error (RMSE) of only 9.0 meV, but is significantly smaller (6.0 meV) for energy points below 2.0 eV relative to the H_2_O + Cu(111) asymptote, representing the unprecedented fitting accuracy for PESs of polyatomic-surface reactions. The 6D site-specific calculations were carried out for various azimuthal angles as well, in an attempt to explore whether there exists the similar averaging approach for the azimuthal angle, as the site-averaging approximation for lateral coordinates (*X* and *Y*) that has been verified in gas-surface reactions by our group (details of the PES and time-dependent wave packet calculations are given in the ESI†).

**Fig. 1:**
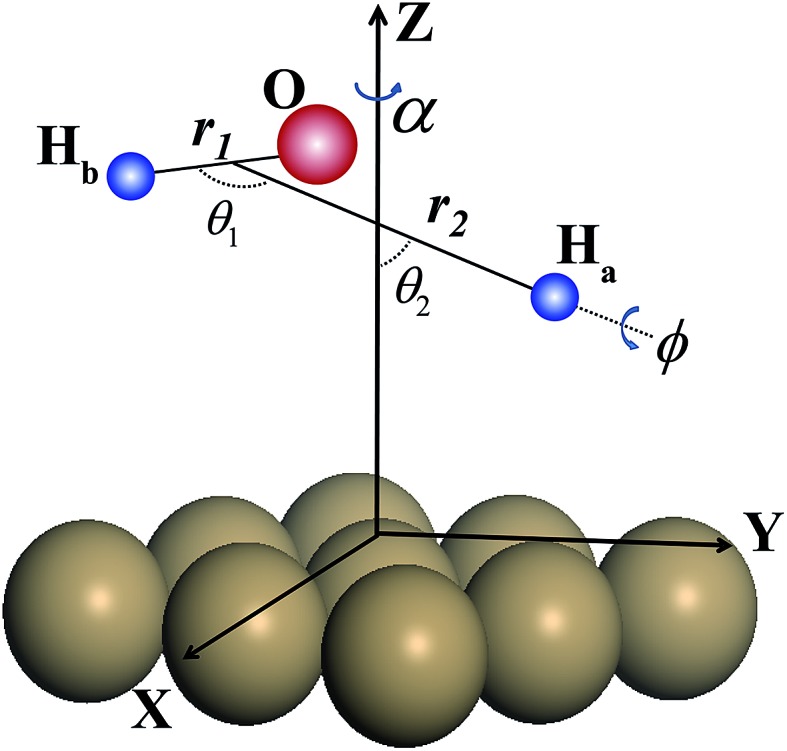
Definition of the molecular coordinates used in studying the dissociative chemisorption of H_2_O on a Cu(111) surface.

Contour plots of the PES as a function of the vertical coordinate *Z* and the distance between the dissociating H atom and the center of mass of nondissociated OH *r*_1_ are illustrated in Fig. 2(a)–(d) for dissociations fixed at the transition state (TS), top, bridge and hcp sites, respectively, with other coordinates fixed at the corresponding saddle-point geometries, which are also shown in the inset. Overall, these fixed-site L-shape reaction paths are quite smooth. The optimized transition-state (TS) geometry by DFT calculations for the dissociation moves towards the hcp site, with one of the O–H bonds stretching out to 1.51 Å and the other keeping around the equilibrium value of 0.982 Å, which are in accord with results obtained on the PES. As shown, the locations of the saddle points on the four impact sites indicate that the dissociation of H_2_O on Cu(111) is a typical late barrier reaction. The static barrier heights for the fixed TS, top, bridge and hcp sites are 1.08 eV, 1.17 eV, 1.13 eV and 1.09 eV, respectively, suggesting that dissociation on the fixed TS site and hcp site is more favorable. Because the overall behavior of the contour plots for the fixed fcc site is nearly the same as that for the hcp site despite the slightly higher (0.01 eV) barrier height of the fcc site, we do not present the results for the fcc site here.

**Fig. 2:**
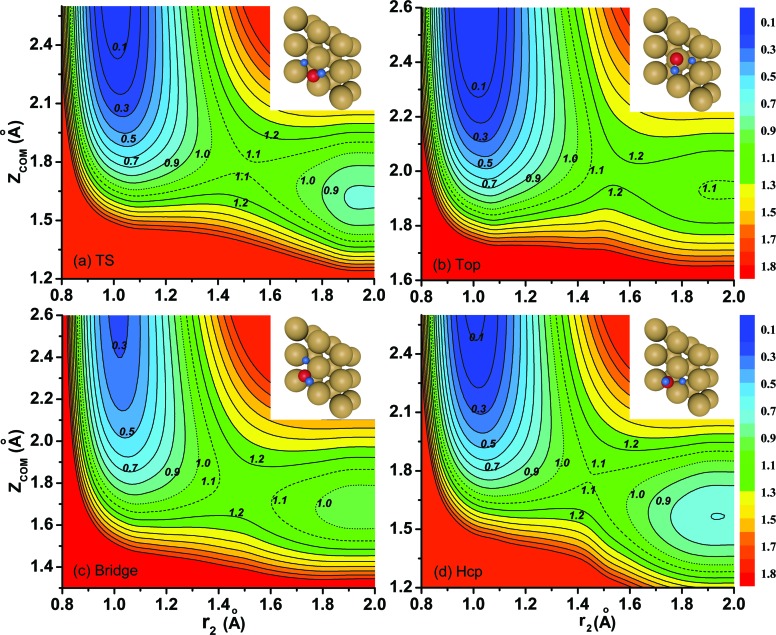
(a)–(d) Fixed-site contour plots of the PES as a function of the vertical distance of H_2_O (*Z*_com_) and the distance between the dissociating H atom and the center mass of OH (*r*_2_), with other coordinates fixed at the corresponding saddle-point geometries. The saddle point geometries are inserted in the right upper corner.

Based on the newly constructed 9D PES, we carried out the first 7D time-dependent wave packet study for the dissociative chemisorption of H_2_O on Cu(111), with the translational coordinates of the H_2_O moiety (*X* and *Y*) fixed at a specific site of impact. These calculations give an excellent opportunity to quantitatively investigate the influence of the azimuthal angle on the dissociation probabilities and the validity of the six-dimensional quantum model, which neglects two lateral surface coordinates and the azimuthal angle as was done previously.^39^–^41^

Fig. 3 shows the seven-dimensional dissociation probabilities for H_2_O initially in the ground rovibrational state for the fixed TS, top, bridge and hcp sites, together with the six-dimensional results with their azimuthal angles *α* fixed at the corresponding saddle-point geometries. The 7D dissociation probability for the top site is about 10 times smaller than those for the other sites, although the static barrier height for the top site is just slightly higher than those for the other sites, indicating the significance of the dynamical investigations as has been found in diatomic molecule–surface reactions.^27^–^29^ On the whole, the 7D and 6D dissociation probabilities increase steadily with increasing kinetic energies; however, significant differences are seen between the 7D and 6D results. The 6D dissociation probabilities with fixed azimuthal angles are all considerably larger than the 7D results in the whole energy region, in particular for the TS site and hcp site. As seen, the 7D probabilities for the TS site and hcp site are roughly 2.5 times and twice as small as the corresponding 6D probabilities at a kinetic energy of 2.0 eV, but these factors become much larger in low energy regions. Similar trends are also found for the other two fixed sites, with the 6D probabilities larger than the 7D probabilities by roughly 30% and 70% at a kinetic energy of 2.0 eV, respectively, for the top and bridge sites.

**Fig. 3:**
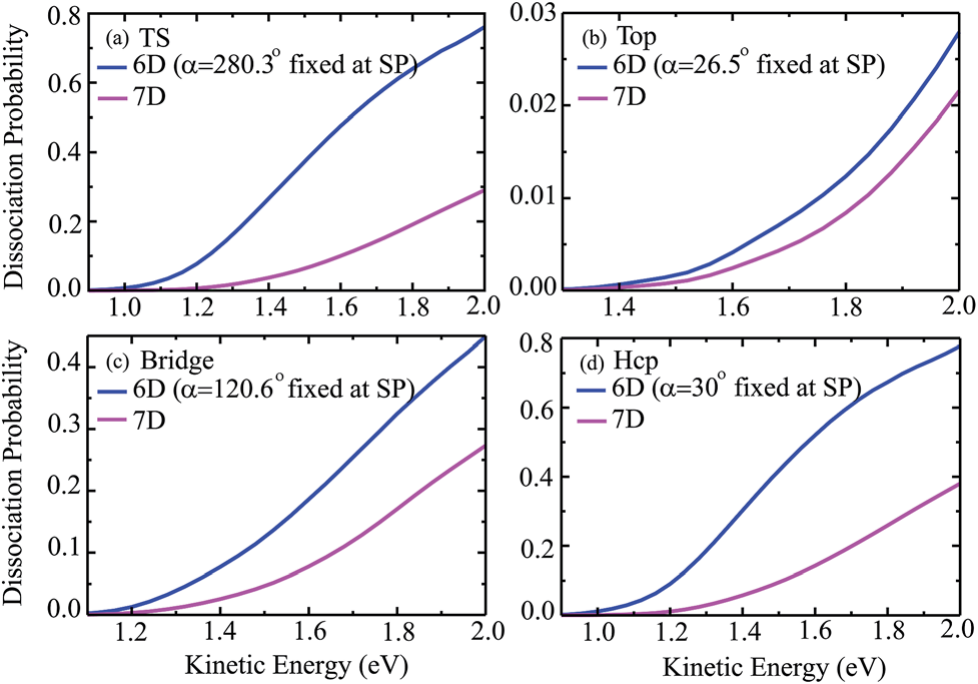
Comparisons of 7D dissociation probabilities and 6D dissociation probabilities with the azimuthal angle *α* fixed at the saddle-point geometry for the fixed TS (a), top (b), bridge (c) and hcp (d) sites, with H_2_O initially in the ground rovibrational state.

To show more clearly the comparison of 6D and 7D dissociation probabilities in very low energy regions below the classical barrier height, we depicted the corresponding results in a logarithmic scale down to 10^–6^ in Fig. 4. We can see obviously the differences between the 6D and 7D results in very low energy regions become more significant, with the 7D dissociation probabilities generally smaller than the corresponding 6D results by a factor of around 9. As a result, the six-dimensional model neglecting the azimuthal angle in quantum dynamical calculations may not reach the quantitative accuracy as compared with the seven-dimensional results.

**Fig. 4:**
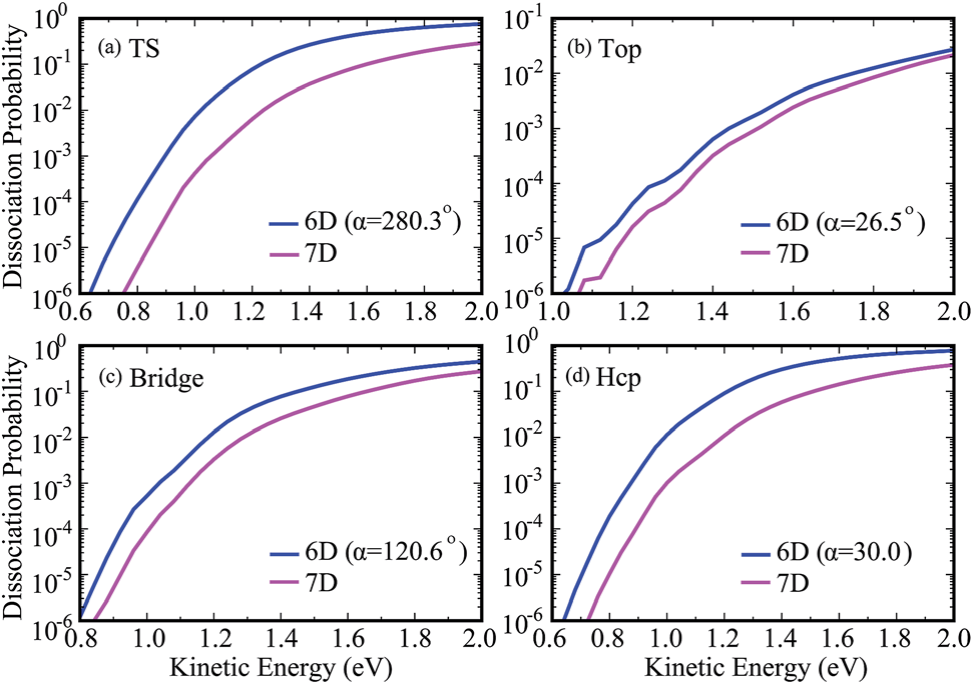
Comparisons of 7D dissociation probabilities and 6D dissociation probabilities down to 10^–6^ in a logarithmic scale.

To better understand the influence of azimuthal angles on the dynamical results for the title molecule–surface reaction, we present in Fig. 5(a)–(d) the static barrier heights for different azimuthal angles on the PES at fixed TS, top, bridge and hcp sites, respectively. The site-specific barrier heights for the azimuthal angle *α* varying from 0° to 360° with an interval of 10°, with other coordinates fixed at the corresponding saddle-point geometries are given. Overall, the barrier heights as a function of azimuthal angle oscillate with some periodic properties due to the symmetry of the PES. The barrier heights for the TS, bridge and hcp sites considerably change with varying azimuthal angles, with the smallest magnitude just above 1.0 eV and largest one around 2.5 eV. In contrast, as shown in Fig. 5(b), the barrier height for the top site fluctuates very slightly with the azimuthal angle and nearly maintains a magnitude of around 1.2 eV in the entire angle region. It is interesting then to see the site-specific dissociation probabilities resulting from different azimuthal angles.

**Fig. 5:**
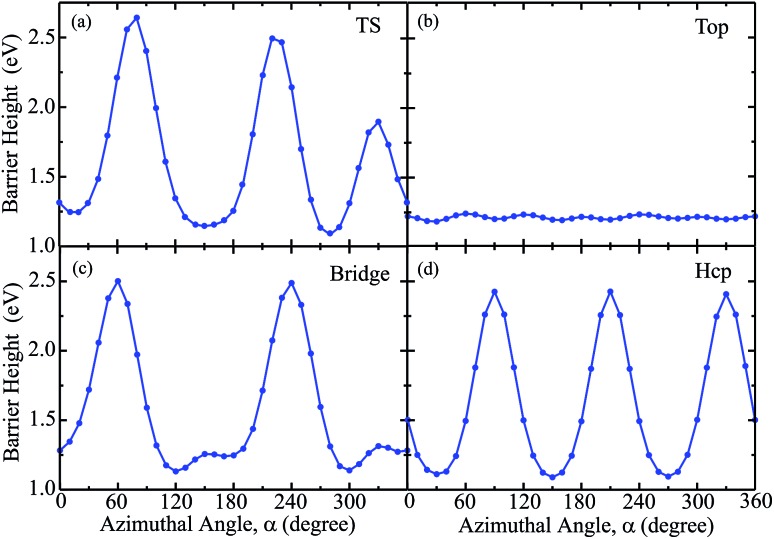
The barrier heights of different azimuthal angles ranging from 0°to 360° with an interval of 10°, with other coordinates fixed at the saddle-point geometry for the fixed TS (a), top (b), bridge (c) and hcp (d) sites.

Fig. 6 shows the six-dimensional dissociation probabilities for several fixed azimuthal angles as well as the seven-dimensional results for H_2_O initially in the ground rovibrational state at the four sites. The azimuthal angles *α* are selected in the range of [230°, 280°], [60°, 30°], [60°, 120°] and [90°, 30°], respectively, for the TS, top, bridge and hcp sites, which correspond to the order of decreasing barrier heights, as shown in Fig. 5. As expected, we can see large differences between the 6D dissociation probabilities with various fixed azimuthal angles and 7D dissociation probabilities. As seen from Fig. 6(a) for the TS site, the 6D dissociation probability increases gradually and the threshold shifts to lower energy as the azimuthal angle *α* increases from 230° to 280°, which corresponds to the barrier height decreasing from roughly 2.5 eV to 1.1 eV in Fig. 5(a). In addition, the magnitude of dissociation probability varies from roughly 0.04 to 0.76 at a kinetic energy of 2.0 eV, differing by a factor of 18 times, which implies that the differences among 6D dissociation probabilities with different azimuthal angles can be very significant, probably due to the large differences of barrier heights. The results for the bridge and hcp sites shown in Fig. 6(c) and (d) demonstrate similar features, indicating that the 6D results depend strongly on the azimuthal angle. However, the 6D dissociation probabilities for the top site in Fig. 6(b) do not change that much as compared with the other three sites, with the magnitude varying from 0.016 to 0.026 at a kinetic energy of 2.0 eV. This is quite understandable as the differences of barrier heights with different azimuthal angles for the top site are very small, as shown in Fig. 5(b). Interestingly, the magnitudes of the 7D dissociation probabilities for the four fixed sites are close to their corresponding medium magnitudes in the 6D results. Then an intriguing question is whether we can find a similar averaging approach for the azimuthal angle in the H_2_O/Cu(111) system, *i.e.*, accurately reproducing the 7D dissociation probability by averaging over the 6D azimuthal angle-fixed results, as has been verified in the site-averaging approach for the surface lateral coordinates (*X* and *Y*) in diatomic molecule–surface reactions.^27^–^29^

**Fig. 6:**
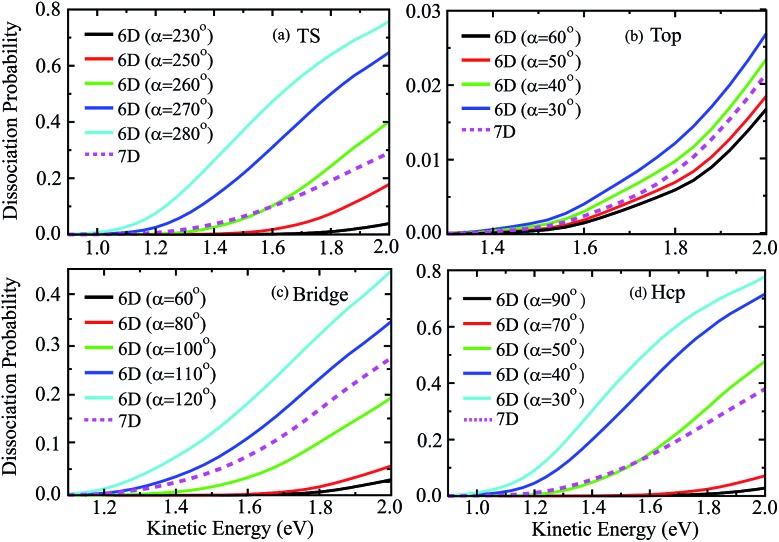
The 6D dissociation probabilities with various fixed azimuthal angles, together with the 7D probabilities for the fixed TS (a), top (b), bridge (c) and hcp (d) sites.

The 6D azimuthal angle-averaged dissociation probabilities are compared with the 7D dissociation probabilities for H_2_O initially in the ground rovibrational state at the fixed TS, top, bridge and hcp sites in Fig. 7. As shown, the 18 angle- and 36-angle averaged results are basically identical, indicating that the 6D angle-averaged results have already converged with the 18 angles included in the averaging, ranging from 0° to 360°. It is interesting that the overall behavior of the angle-averaged dissociation probabilities resemble those of the 7D dissociation probabilities. Compared with the 6D dissociation probabilities with the azimuthal angle fixed at the corresponding saddle-point geometries, also shown in the blue curves in Fig. 7, the agreement between the angle-averaged and the 7D dissociation probabilities is significantly improved.

**Fig. 7:**
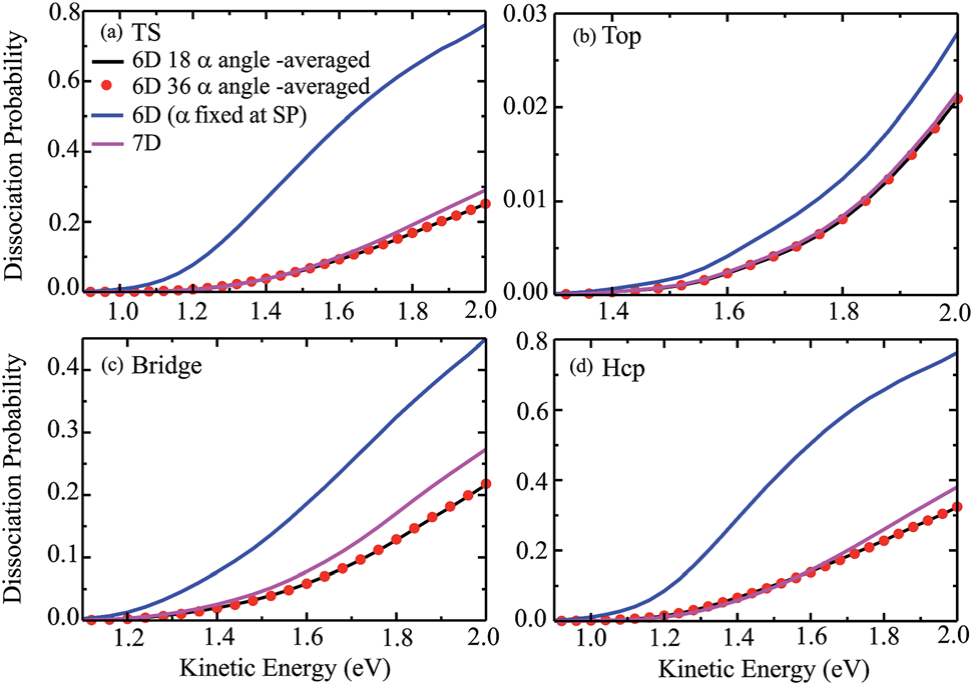
The angle-averaged dissociation probabilities by averaging the 6D results over 18 and 36 azimuthal angles, 6D dissociation probabilities with the azimuthal angle fixed at the saddle-point geometry, and 7D dissociation probabilities for the fixed TS (a), top (b), bridge (c) and hcp (d) sites.

In particular for the top site, as shown in Fig. 7(b), the agreement between the angle-averaged dissociation probabilities and the 7D results is excellent and impressive in the entire kinetic energy region of [1.3, 2.0] eV. For the TS and hcp sites, the angle-averaged results can accurately reproduce the 7D results as well, except for a kinetic energy higher than 1.6 eV. The angle-averaged results become slightly smaller than the 7D results when the kinetic energy exceeds 1.6 eV, but the agreement between them is still good. Although this agreement for the bridge site is less satisfactory, with the angle-averaged probabilities smaller than the 7D results by a factor of roughly 18%, the former can reproduce the latter in the threshold region. The excellent agreement achieved between the 6D angle-averaged and 7D probabilities for the top site results from the relatively weak dependence of the 6D probabilities on azimuthal angles, as discussed above. Overall, the 7D dissociation probabilities for fixed sites can be well reproduced by averaging the 6D results over 18 azimuthal angles, in particular in low kinetic energy regions.

To summarize, in this study we carried out the first seven-dimensional quantum dynamics calculations for the dissociative chemisorption of H_2_O on a rigid Cu(111) surface with H_2_O fixed at TS, top, bridge and hcp sites, based on an accurate full-dimensional (9D) PES newly developed by neural network fitting to roughly 80 000 DFT points. The current 7D quantum dynamical model with one more degree of freedom coupled, *i.e.*, the azimuthal angle, represents much more computational effort compared with the previous 6D quantum model which neglects the azimuthal angle and surface lateral coordinates. It gives a good chance to quantitatively investigate the dependence of azimuthal angles on the dissociation probabilities, as well as to identify the quantitative relationship between the 7D and 6D dissociation probabilities. The calculated 7D dissociation probabilities at the TS, top, bridge and hcp sites are quite different from the corresponding 6D probabilities with various fixed azimuthal angles, indicating the great importance of the 7D quantum dynamical investigations. A new azimuthal angle-averaging approach is proposed that one can accurately reproduce the 7D probability by averaging the 6D azimuthal angle fixed probabilities over 18 or 36 angles, ranging from 0° to 360°, in particular in low kinetic energy regions, because very satisfactory agreement has been achieved between the 7D probabilities and the 18/36 azimuthal angle-averaged 6D probabilities for H_2_O initially in the ground rovibrational state. The validity of this new approach for excited rovibrational states of H_2_O will be verified in the near future. Furthermore, in principle we are capable of calculating 7D dissociation probabilities for many sites of impact, which can be employed in the site-averaging approach to accurately approximate the full-dimensional (9D) dissociation probability for the dissociative chemisorption of H_2_O on Cu(111).

## Supplementary Material

Supplementary information
