# Supplementary material for: A seven-dimensional quantum dynamics study of the dissociative chemisorption of H2O on Cu(111): effects of azimuthal angles and azimuthal angle-averaging
Source: Chem Sci. 2015 Nov 25;7(3):1840–5. doi: 10.1039/c5sc03689e (PMC5965058; doi:10.1039/c5sc03689e)
Supplement: Supplementary file 1 [file SC-007-C5SC03689E-s001.pdf]

Electronic Supplementary Material (ESI) for Chemical Science.
This journal is © The Royal Society of Chemistry 2015

Supplementary Information

A seven-dimensional quantum dynamics study of the dissociative chemisorption of H_2O on Cu(111): Effects of azimuthal angles and azimuthal angle-averaging

Tianhui Liu[a,b], Zhaojun Zhang[b], Bina Fu[b], Xueming Yang[a,b] and Dong H. Zhang[b]

[a]*Department of Chemical Physics, University of Science and Technology of China, Hefei, P.R. China 230026 and*

[b]*State Key Laboratory of Molecular Reaction Dynamics and Center for Theoretical and Computational Chemistry, Dalian Institute of Chemical Physics, Chinese Academy of Sciences, Dalian, P.R. China 116023*

PACS numbers:

I. 9D PES

All the planewave DFT calculations were carried out with the Vienna *ab initio* simulation package (VASP)[1, 2]. The interaction between ionic cores and electrons was described by fully nonlocal optimized projector augmented-wave (PAW) potentials[3], and the Kohn-Sham valence electronic states were expanded in a planewave basis set[4]. The electron exchange correlation effects were treated within the generalized gradient approximation (GGA)[5], using the Perdew-Wang (PW91) functional[6]. The Cu(111) substrates consist of four layers with a 2×2 surface unit cell(1/4-ML coverage). A vacuum region between two repeated cells is 12 Å, the Monkhorst-Pack k-points grid mesh is 5×5×1[7] and the planewave expansion is truncated at the kinetic energy of 400 eV. The optimized lattice constants for bulk Cu is 3.636 Å in this work, which agrees well with the experimental value (3.615 Å)[8]. To validate our slab model, we calculate the relative barrier energy E_b and the relative product energy E_p with different computational details. The relative barrier energy E_b and the relative product energy E_p are defined as $E_b = \epsilon_b - \epsilon_\infty$ and $\epsilon_p - \epsilon_\infty$ where ϵ_b, ϵ_p, ϵ_∞ are, respectively, the absolute barrier, product, and asymptotic energies. The detailed parameters used in the DFT calculations are shown in Table I, with the cutoff kinetic energies ranging from 300 eV to 450 eV, the number of layers ranging from four to six, the Monkhorst-Pack k-points grid mesh from 3×3×1 to 8×8×1 and the size of the supercell ranging from 2×2 to 4×4. We can see the barrier height E_b with four layers is very close to that with six layers and E_b just varies slightly with the increasing size of supercell. Although the product energy E_p has larger variations and seems difficult to converge, the adsorption energy and activation energy calculated based on the current slab model are -0.19 eV and 1.21 eV, which agree well with the recent work by Jiang and Guo for the title system (-0.17eV and 1.22 eV)[9]. The two energies are also consistent with previous studies for water dissociation on Cu(111) using different computational details including higher numbers of layers and larger supercell[10–13]. Due to the large number of the energy points we needed to obtain an accurate PES for this system, the moderate slab model was found to be the best compromise between efficiency and accuracy and was used for the computation of the PES.

The climbing-image nudged elastic band (CI-NEB) method was used to determine the transition state (TS) and minimum energy path [14, 15]. Our calculations are considered to converge when all forces are smaller than 0.01 eV/Å. We employed the feed forward neural

networks (NN) to fit the full-dimensional PES with two hidden layers connecting the input layer and output layer, denoted as $I-J-K-1$ NN. It has I nodes in the input layer, which equals to the number of input Cartesian coordinates employed here for a molecular configuration, and one nodes in the output layer corresponding to the potential energy value of the input configuration. More details of the NN method to fit the PES can be found elsewhere[16–18].

A total of 81102 DFT energy points were calculated to be used in the fitting. All those molecular configurations are mapped in the irreducible triangle of the surface unit cell (p3m1 plane group symmetry) which is spanned by the top, hollow and fcc sites[19], as shown in Fig. S1. Considering the continuity of the boundary of irreducible triangle, the data size was enlarged to 93908 energy points using the method we have used in the HCl/Au(111)system[16] and was included in fitting the PES of the title molecule-surface interaction system. To accelerate the NN fitting procedure, we divided the data points into four parts: entrance part (I), interaction parts (II and III), and exit part(IV), with some overlaps between I and II parts, II and III parts, III and IV parts as shown in TABLE II. Less neurons are required to fit these parts separately to reach a desired root mean square error(RMSE), leading to a much more efficient fitting without any loss of accuracy. The same fitting procedure has been used successfully in gas phase reactions like H+H_2O and H+CO_2[17, 18], and the gas-surface reaction HCl+Au(111)[16]. We used the 9 elements of Cartesian coordinates of H_2O as the input coordinates for the NN fit

Convergence properties of these parts are tested independently with more effort paid to the important regions of a PES. For each part we obtained three fits with small RMSEs using different NN architectures or the same architecture with differen weights and biases, as shown in Table III. We can see that the differences of the RMSEs between current three fits in each part are small (approximately 1 meV), with the maximal difference of 1.1 meV for the RMSEs of the three fits in part II, ranging from 9.2 to 10.3 meV. The combination of the 12 segmental fits shown in Table II results in a total of 81 global PESs, one of which is denoted as the NN1 PES here with the NN structures of 9-80-75-1, 9-80-75-1, 9-80-75-1 and 9-85-70-1, and RMSEs of 3.2 meV, 10.1 meV, 9.8 meV and 13.1 meV for part I, II, III and IV, respectively. The overall RMSE is only 9.0 meV, but significantly smaller (6.0 meV) for energy points below 2.0 eV relative to the H_2O + Cu(111) asymptote. The seven-dimensional dissociation probabilities for H_2O in the ground rovibrational state at the

fixed bridge and top sites were calculated on the three global PESs which were randomly selected from resulting 80 PESs except NN1. These probability curves are compared with that calculated on NN1 in Fig. S2, in the kinetic energy region between 0.8 eV and 2.0 eV. As displayed in Fig. S2 (a) and (b), the dissociation probability is a monotonically increasing function as the kinetic energy. The probabilities obtained on the three PESs randomly selected are essentially identical to that on the NN1 PES, indicating that the PES is well converged with the fitting procedure. Besides, we note that the global NN1 PES is produced by connecting the four segmental parts with smooth switching functions. The convergence for the continuity when crossing the boundary of each fit was also checked using the TDWP calculations. It might introduce some errors in quasiclassical trajectory (QCT) calculations when a trajectory passes through the boundary due to the calculations of derivatives, but this is not a problem for quantum dynamics. The NN1 PES is truncated at r_1=1.9 Å, which is reasonable as the O-H bond length at the TS geometry is only 1.5 Å. In addition, the dissociation probabilities obtained on the PES with r_1 truncated at 1.9 Å are the same as those with r_1 truncated at 2.3 Å.

The minimum energy path of the dissociative chemisorption of H_2O on Cu(111) obtained by the CI-NEB method is shown in Fig. S3, with energies relative to the reactants H_2O + Cu(111) asymptote. This path was discretized by eight images between the reactant and product. The reactant H_2O is 6.0 Å above the top site of the Cu(111) surface, and is almost parallel to the surface. There exists a van der waals well of about -0.13 eV, where the two O-H bonds nearly maintain their equilibrium distances (0.98 Å) and the COM of H_2O is 2.50 Å above the surface, but it moves slightly away from the top site. The H_2O molecule at the optimized transition state (TS) moves towards the hcp site, with one of the O-H bond lengths stretched out to 1.51 Å and the other keeps around the equilibrium value of 0.982 Å. The structure parameters optimized at the transition state is X=2.133 Å, Y=0.969 Å, Z=1.772 Å, r_1=0.982 Å, r_2=1.511 Å,θ_1 =117.3°,θ_2 =66.1° ϕ=306.4°,α =160.3°and the barrier height is 1.083 eV, which agrees well with the transition state geometry obtained from the fitted NN1 PES of X=2.133 Å, Y=0.968 Å, Z=1.772 Å, r_1=0.982 Å, r_2=1.521 Å,θ_1 =119.9°,θ_2 =65.5° ϕ=307.1°,α =160.3° with the barrier height of 1.084 eV. The product configuration corresponds to the position where OH is adsorbed on a fcc site, and H is adsorbed on a nearest-neighbor fcc site. The minimum energy paths calculated from the fitted NN1 PES and the DFT method at the same geometries are also compared and shown

in Fig. S4. As we can see, the very good agreement is achieved. Therefore, the current NN1 PES constructed using NN for the dissociative chemisorption of H_2O on Cu(111) is sufficiently accurate to be used in investigating the quantum reaction dynamics and we chose it as the final PES.

Finally, It is worthwhile to discuss the anisotropy problem for the asymptotic region of the PES in the slab model[20–22]. In our earlier work of developing the PES of the HCl/Au system[16], we did refine the original PES by combining the gas-phase HCl potential and the asymptotic gas-surface PES using a switching function along the Z coordinate, as was done by Kroes and coworkers in the CH_4/Ni(111) system[21]. It turns out the differences between the computed dissociation probabilities of HCl/DCl+Cu(111) on the modified and original PESs are very small. Recently, Guo and coworkers used the similar switching function to refine their 6D PES for the title H_2O+Cu(111) reaction. Very small differences can be seen between the results on the original and modified PESs[22]. Thus, we did not use the switching function to modify our NN1 PES, though it may introduce slightly quantitative differences in calculating dissociation probabilities, which should not affect the main conclusions drawn in the main text as we expect.

II. TDWP APPROACH

The fully coupled quantum dynamics calculations for the dissociative chemisorption of H_2O on a corrugated, rigid metal surface should include nine degrees of freedom (9D). Here, we fixed the center of mass (COM) of the H_2O moiety on a specific site of impact of the Cu(111) surface. Therefore, the 7D Hamiltonian for the title reaction is expressed in terms of molecule coordinates (Z, r_1, r_2, θ_1, θ_2 , ϕ, α) (shown in Fig. 1) as,

$$\hat{H} = -\frac{1}{2M}\frac{\partial^2}{\partial Z^2} - \frac{1}{2\mu_1}\frac{\partial^2}{\partial r_1^2} - \frac{1}{2\mu_2}\frac{\partial^2}{\partial r_2^2} + \frac{j^2}{2\mu_1 r_1^2} + \frac{L^2}{2\mu_2 r_2^2} + V(Z, r_1, r_2, \theta_1, \theta_2, \phi, \alpha) \quad (1)$$

where M is the mass of H_2O, μ_1 is the reduced mass of diatom OH_b , μ_2 is the reduced mass of diatom OH_b and atom H_a, r_1 is the bond length of non-dissociative OH_b bond, r_2 is the distance from the COM of diatom OH_b and atom H_a, Z is the vertical distance between the COM of H_2O and the surface, j is the rotational angular momentum operator for diatom OH_b and L is the orbital momentum operator of H_a with respect to diatom OH_b. The last term $V(Z, r_1, r_2, \theta_1, \theta_2, \phi, \alpha)$ is the interaction potential energy.

The time-dependent wave function is expanded in terms of translational basis of Z, $\phi_{v_1}(r_1)$, $\phi_{v_2}(r_2)$and angular momentum eigenfunctions $Y_{jL}^{JM}(Z, r_1, r_2)$ as

$$\Psi(Z, r_1, r_2, \theta_1, \theta_2, \phi, \alpha, t) = \sum_{n,v,j,L,M} F_{nvjLM}(t) u_n^v(Z) \phi_{v_1}(r_1) \phi_{v_2}(r_2) Y_{jL}^{JM}(Z, r_1, r_2) \quad (2)$$

where u_n^v is the translational basis function for Z which is dependent on v as given in Ref[23].

The total angular momentum basis $Y_{jL}^{JM}(Z, r_1, r_2)$ in Eq. (2) are defined as

$$Y_{jL}^{JM}(Z, r_1, r_2) = \sum_K D_{MK}^{J*}(\phi, \theta_2, \alpha) \sqrt{\frac{2L+1}{4\pi}} \times \langle jKL0|JK\rangle y_{jK}(\theta_1, 0) \quad (3)$$

where $D_{MK}^{J*}(\phi, \theta_2, \alpha)$ is the Wigner rotation matrix and y_{jK} are spherical harmonics. K is the projection of J(rotational angular momenta of H_2O) onto the H_a-OH_b axis. M is the projection of J on the surface normal which is conserved in six-dimensional calculations.

The wave function is propagated using the split-operator method[24] and the time-dependent wave function is absorbed at the edges of the grid to avoid boundary reflections[25].

The initial state-selected total dissociation probability is obtained by projecting out the energy dependent reactive flux. If ψ_{iE}^+ denotes the time-independent (TI) full scattering wave function, where i and E are, respectively, initial state and energy labels, the total reaction probability from an initial state i can be obtained by the formula

$$P_i^R = < \psi_{iE}^+ \mid \hat{F} \mid \psi_{iE}^+ >, \quad (4)$$

In the above equation, $\hat{F}$ is the flux operator, defined as

$$\hat{F} = \frac{1}{2}[\delta(\hat{s} - s_0)\hat{v}_s + \hat{v}_s \delta(\hat{s} - s_0)] \quad (5)$$

where s is the coordinate perpendicular to a surface located at s_0 for flux evaluation, and $\hat{v}_s$ the velocity operator corresponding to the coordinate s. The full TI scattering wave function is normalized as $< \psi_{iE}^+ \mid \psi_{iE'}^+ >= 2\pi\delta(E - E')$. Using the expression in (5), Eq.(4) can be simplified to yield

$$P_i(E) = \frac{\hbar}{\mu_2} \mathrm{Im}(\left\langle \psi_{iE}^+ | \psi_{iE}^{+'} \right\rangle)|_{s=s_0}, \quad (6)$$

where

$$\left|\psi_{iE}^+\right\rangle = \frac{1}{a_i(E)} \int_0^\infty e^{i(E-H)t/\hbar} \left|\Psi_i(0)\right\rangle dt, \quad (7)$$

with $a_i(E) = \langle \phi_{iE} | \Psi_i(0) \rangle$ being the overlap between the initial wave packet $\Psi_i(0)$ and the energy-normalized asymptotic scattering function ϕ_{iE}.

The numerical parameters used in the 7D calculations are as follows: We used 311 and 30 sin-type DVR[26, 27] points to describe Z and r_2 coordinates, ranging from 2.0 to 14.5 bohrs and 1.2 to 5.5 bohrs, respectively. The non-reactive r_1 coordinate was described with 3 PODVR points, between 1.2-3.5 bohrs. The j_{max}, L_{max}, K_{max} and M_{max} equals 20, 25, 20 and 10, respectively. The imaginary absorbing potentials are placed in the range of Z between 12.5 and 14.5 bohrs and r_2 between 4.0 and 5.5 bohrs, respectively, and the dissociation flux is calculated on the dividing surface of r_2=3.5 bohr. The time step for the propagation is 10 a.u. and we propagate the wave packets for 12000 a.u. of time to converge the dissociation probabilities. Due to the non-reactive of one OH bond in current theory, the computed dissociation probabilities were multiplied with a factor of 2 which correspond to the results for two reactive bonds in this paper. The input parameters of the 6D calculations are the same as those of the 7D calculations, except that M_{max} is conserved to 0 with the azimuthal angle fixed, the number of PODVR points for the r_1 coordinate increases to 4, and L_{max} equals 30. In addition, the parameters used are all checked to converge well by the TDWP calculations for all sites. We noted the 6D parameters in some coordinates (r_2 and θ_2) are slightly larger than those of 7D calculations, which is reasonable because the parameters used in reduced-dimensional calculations are generally larger than those in higher or full-dimensional calculations. Similar behavior can be seen in the HCl/DCl+Au(111) systems [28, 29]. The NN1 PES is employed in dynamics calculations and all the dynamics results in this article are based on the NN1 PES.

---

[1] G. Kresse and J. Furthmuller, *Comp. Mater. Sci.*, 1996, **6**, 15–50.

[2] G. Kresse and J. Furthmller, *Phys. Rev. B*, 1996, **54**, 11169–11186.

[3] P. E. Blöchl, *Phys. Rev. B*, 1994, **50**, 17953–17979.

[4] G. Kresse and D. Joubert, *Phys. Rev. B*, 1999, **59**, 1758–1775.

[5] J. P. Perdew, K. Burke and M. Ernzerhof, *Phys. Rev. Lett.*, 1996, **77**, 3865–3868.

[6] J. P. Perdew, K. A. Jackson, M. R. Pederson, D. J. Singh and C. Fiolhais, *Phys. Rev. B*, 1992, **46**, 6671–6687.

[7] H. J. Monkhorst and J. D. Pack, *Phys. Rev. B*, 1976, **13**, 5188–5192.

[8] *CRC Handbook of Chemistry and Physics*, ed. W. M. Haynes., CRC Press, New York, 2013.

[9] B. Jiang, X. Ren, D. Xie and H. Guo, *Proc. Natl. Acad. Sci. U. S. A.*, 2012, **109**, 10224–10227.

[10] Q.-L. Tang, Z.-X. Chen and X. He, *Surf. Sci.*, 2009, **603**, 2138–2144.

[11] A. A. Phatak, W. N. Delgass, F. H. Ribeiro and W. F. Schneider, *J Phys. Chem. C*, 2009, **113**, 7269–7276.

[12] X.-K. Gu and W.-X. Li, *The J. Phys. Chem. C*, 2010, **114**, 21539–21547.

[13] A. A. Gokhale, J. A. Dumesic and M. Mavrikakis, *J. Am. Chem. Soc.*, 2008, **130**, 1402–1414.

[14] G. Henkelman, B. P. Uberuaga and H. Jónsson, *J. Chem. Phys.*, 2000, **113**, 9901–9904.

[15] *Nudged elastic band method for finding minimum energy paths of transitions. Classical and Quantum Dynamics in Condensed Phase Simulations*, ed. B. J. Berne, G. Ciccotti and D. F. Coker., World Scientific, Singapore, 1998.

[16] T. Liu, B. Fu and D. H. Zhang, *Sci. China Chem.*, 2013, **57**, 147–155.

[17] J. Chen, X. Xu and D. H. Zhang, *J. Chem. Phys.*, 2013, **138**, 221104.

[18] J. Chen, X. Xu and D. H. Zhang, *J. Chem. Phys.*, 2013, **138**, 154301.

[19] T. J. Frankcombe, M. A. Collins and D. H. Zhang, *J. Chem. Phys.*, 2012, **137**, 144701.

[20] H. F. Busnengo, C. Crespos, W. Dong, J. C. Rayez and A. Salin, *J. Chem. Phys.*, 2002, **116**, 9005.

[21] G. P. Krishnamohan, R. A. Olsen, G. J. Kroes, F. Gatti and S. Woittequand, *J. Chem. Phys.*, 2010, **133**, 144308.

[22] B. Jiang, J. Li, D. Xie and H. Guo, *J. Chem. Phys.*, 2013, **138**, 044704(1–9).

[23] B. Fu and D. H. Zhang, *J. Chem. Phys.*, 2015, **142**, 064314.

[24] J. Fleck, J. A., J. R. Morris and M. D. Feit, *Appl. Phys.*, 1976, **10**, 129–160.

[25] D. Neuhasuer and M. Baer, *J. Chem. Phys.*, 1989, **90**, 4351–4355.

[26] D. T. Colbert and W. H. Miller, *J. Chem. Phys.*, 1992, **96**, 1982–1991.

[27] Z. Bacic and J. C. Light, *Annu. Rev. Phys. Chem.*, 1989, **40**, 469–498.

[28] T. Liu, B. Fu and D. H. Zhang, *J. Chem. Phys.*, 2014, **140**, 144701.

[29] T. Liu, B. Fu and D. H. Zhang, *J. Chem. Phys.*, 2013, **139**, 184705.

TABLE I. The relative barrier energy E_b and the relative product energy E_p are compared between different computation details, respectively.

Computation details	E_b	E_p
Four layers, 1/4 ML, 300eV,$5 \times 5 \times 1$ k-points	1.036	0.182
Four layers, 1/4 ML, 350eV,$5 \times 5 \times 1$ k-points	1.066	0.207
Four layers, 1/4 ML, 400eV,$5 \times 5 \times 1$ k-points	1.083	0.225
Four layers, 1/4 ML, 450eV,$5 \times 5 \times 1$ k-points	1.086	0.228
Four layers, 1/4 ML, 400eV,$3 \times 3 \times 1$ k-points	1.057	0.161
Four layers, 1/4 ML, 400eV,$8 \times 8 \times 1$ k-points	1.144	0.303
Five layers, 1/4 ML, 450eV,$5 \times 5 \times 1$ k-points	1.227	0.422
Six layers, 1/4 ML, 450eV,$5 \times 5 \times 1$ k-points	1.087	0.159
Four layers, 1/9 ML, 400eV,$5 \times 5 \times 1$ k-points	1.071	-0.01
Four layers, 1/16 ML, 400eV,$5 \times 5 \times 1$ k-points	1.126	0.119

TABLE II. The range of the four parts divided and the number of energy points calculated in each part (Z is the vertical distance between the COM of H_2O and the surface, r_2 is the distance between the COM of OH and H). The distances are in Å.

	Region	Number of data points
Part I (a)	$Z \geqslant 3.9$	34056
Part II (b)	$Z \leqslant 4.1$ and $r_2 \leqslant 1.15$	9077
Part III (c)	$Z \leqslant 4.1$ and $r_2 \leqslant 1.5$ and $r_2 \geqslant 1.05$	30440
Part IV (d)	$Z \leqslant 4.1$ and $r_2 \geqslant 1.4$	37565

TABLE III. Neural network structure parameters and fitting errors (meV) for the four segmental parts (shown in parentheses). In each part we used different weights and biases to give three fits.

	Fit 1	Fit 2	Fit 3
Part I (a)	9-80-75-1 (3.2)	9-80-75-1 (3.3)	9-90-75-1 (3.8)
Part II (b)	9-80-75-1 (10.1)	9-80-75-1 (9.2)	9-80-75-1 (10.3)
Part III (c)	9-80-75-1 (9.8)	9-80-75-1 (10.2)	9-80-75-1 (10.4)
Part IV (d)	9-85-75-1 (13.1)	9-85-75-1 (13.9)	9-85-75-1 (14.1)

FIGURE CAPTION

Fig. S1 The irreducible triangle of Cu(111) surface unit cell (shown in red triangle)in C_{3v} symmetry. The atoms in the first, second, and third layers are shown in gray, yellow, and black spheres, respectively. Note that the atoms in the third layer are right at the fcc site. Atoms in the fourth (fifth, sixth) layer are directly below the atoms in the first (second, third) layer, respectively.

Fig. S2 The seven-dimensional dissociation probabilities for H_2O initially in the ground rovibrational state at fixed top and bridge sites calculated on four PESs we selected randomly from the total of 81 PESs (a, b, c, and d denote part I, II, III, and IV, respectively).

Fig. S3 Minimum energy pathway for the dissociative chemisorption of H_2O on Cu(111) obtained using CINEB method with eight images.

Fig. S4 Comparison of the fitted NN1 PES and corresponding DFT energies along the minimum energy pathway.

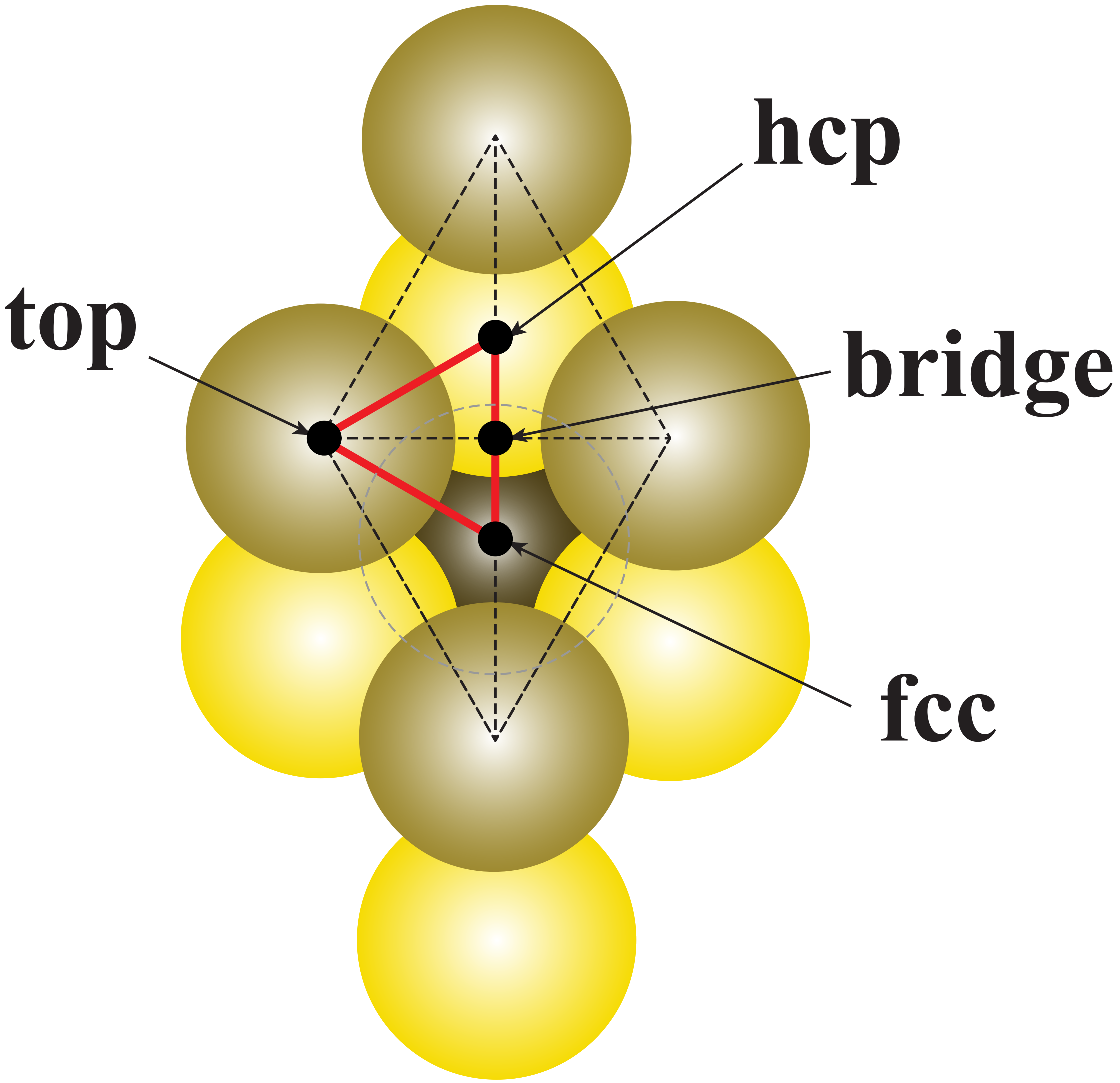
hcp
top
bridge
fcc

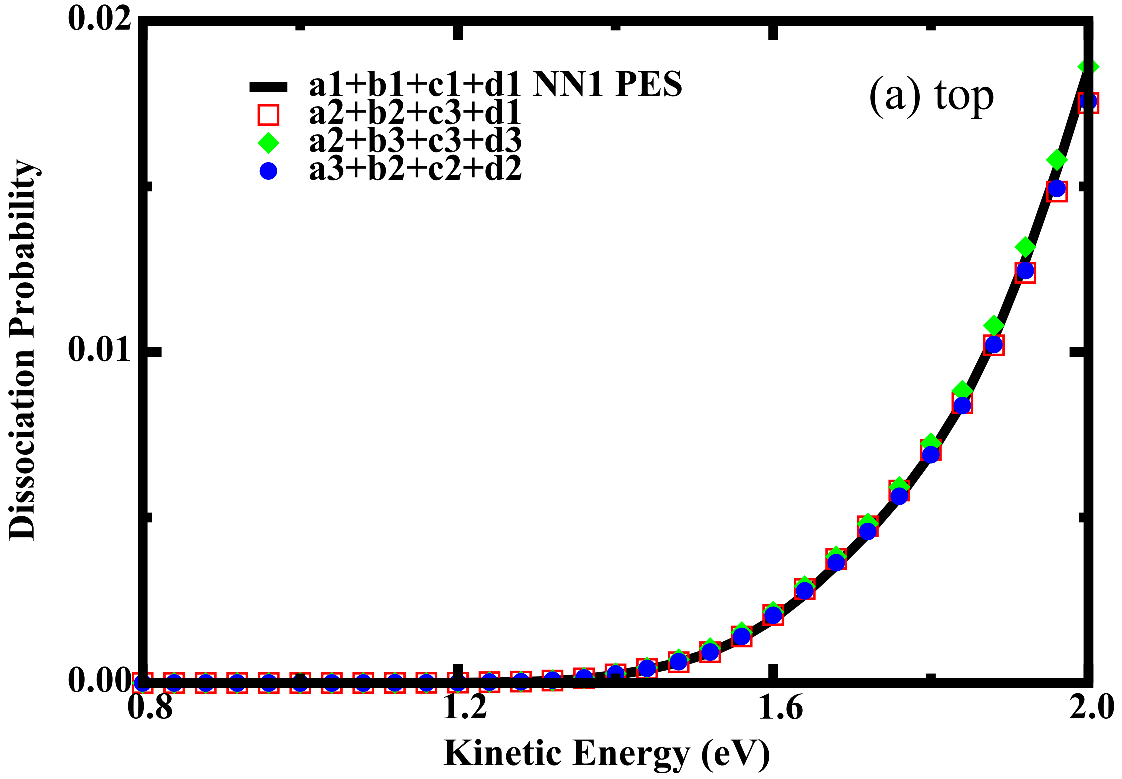
0.02
0.01
0.00
0.8
1.2
1.6
2.0
Dissociation Probability
Kinetic Energy (eV)
a1+b1+c1+d1 NN1 PES
a2+b2+c3+d1
a2+b3+c3+d3
a3+b2+c2+d2
(a) top

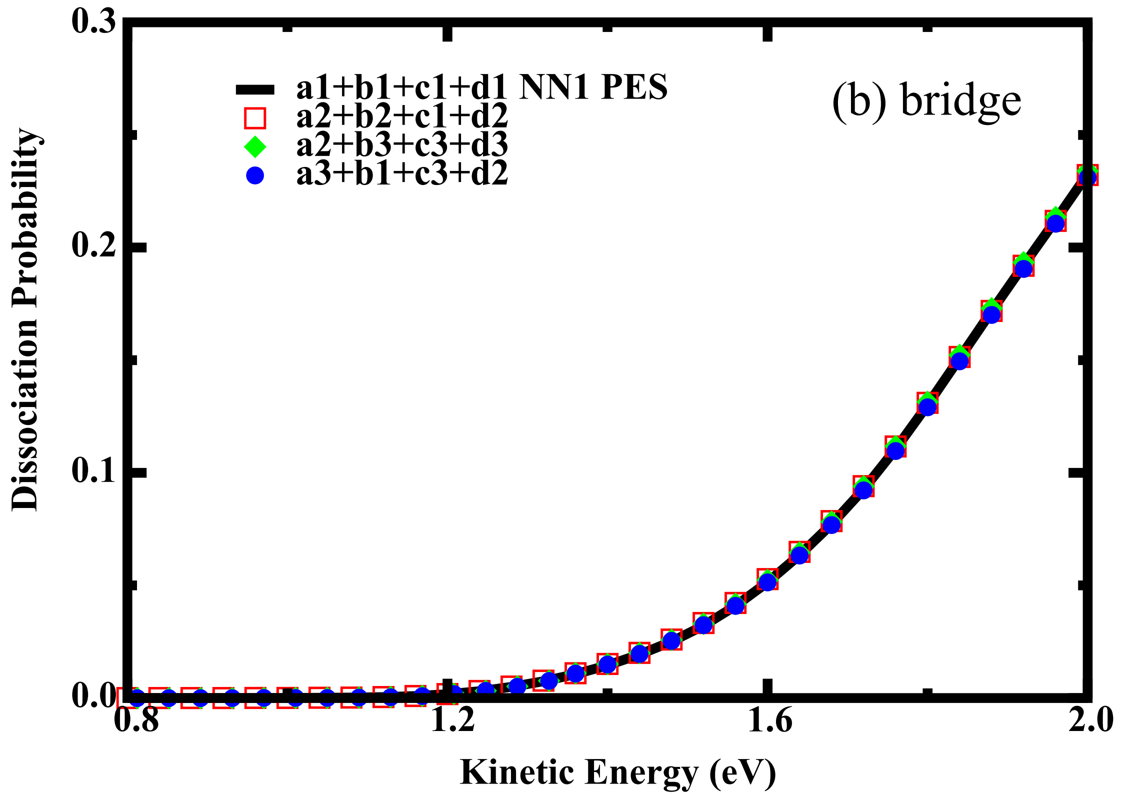
0.3
0.2
0.1
0.0
0.8
1.2
1.6
2.0
Dissociation Probability
Kinetic Energy (eV)
a1+b1+c1+d1 NN1 PES
a2+b2+c1+d2
a2+b3+c3+d3
a3+b1+c3+d2
(b) bridge

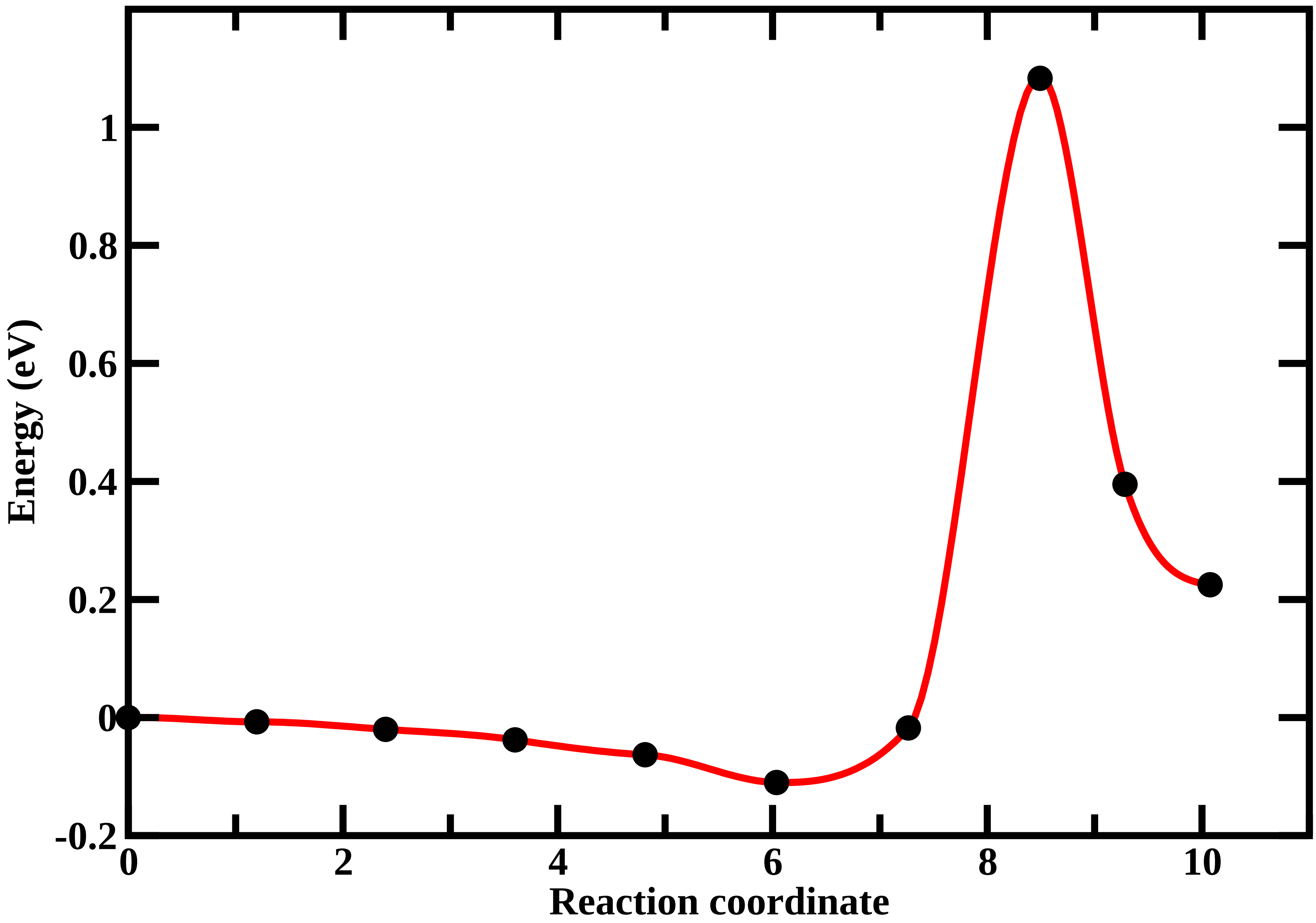

Energy (eV)
1
0.8
0.6
0.4
0.2
0
-0.2
0
2
4
6
8
10
Reaction coordinate

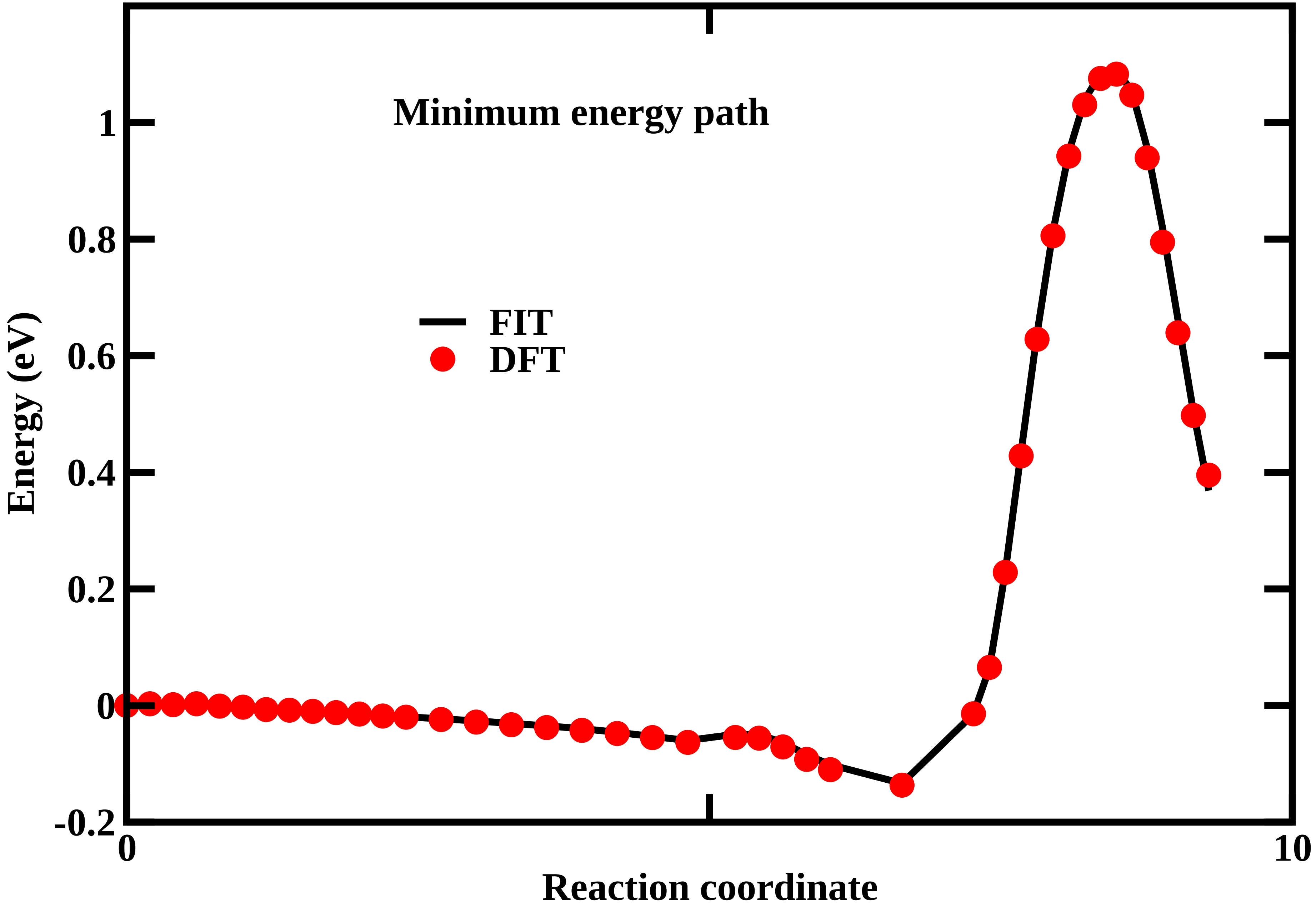

Minimum energy path
FIT
DFT
Energy (eV)
Reaction coordinate
1
0.8
0.6
0.4
0.2
0
-0.2
0
10
